# Mitochondrial DNA heteroplasmy analysis in keratoconus patients from China

**DOI:** 10.3389/fgene.2023.1251951

**Published:** 2023-09-18

**Authors:** Liyan Xu, Kaili Yang, Qi Fan, Yuwei Gu, Shengwei Ren

**Affiliations:** Henan Provincial People’s Hospital, Henan Eye Hospital, People’s Hospital of Zhengzhou University, Zhengzhou, China

**Keywords:** keratoconus, mitochondrial DNA, heteroplasmy, variant, m.16180_16181delAA

## Abstract

**Background:** Mitochondrial DNA (mtDNA) variants have been implicated in keratoconus (KC). The present study aimed to characterize the mtDNA heteroplasmy profile in KC and explore the association of mitochondrial heteroplasmic levels with KC.

**Methods:** Mitochondrial sequencing of peripheral blood samples and corneal tomography were conducted in 300 KC cases and 300 matched controls. The number of heteroplasmic and homoplasmic variants was calculated across the mitochondrial genome. Spearman’s correlation was used to analyze the correlation between the number of heteroplasmic variants and age. The association of mtDNA heteroplasmic level with KC was analyzed by logistic regression analysis. Moreover, the relationship between mitochondrial heteroplasmic levels and clinical parameters was determined by linear regression analysis.

**Results:** The distribution of mtDNA heteroplasmic variants showed the highest number of heteroplasmic variants in the non-coding region, while the *COX3* gene exhibited the highest number in protein-coding genes. Comparisons of the number of heteroplasmic and homoplasmic non-synonymous variants in protein-coding genes revealed no significant differences between KC cases and controls (all *p* > 0.05). In addition, the number of heteroplasmic variants was positively associated with age in all subjects (*r* = 0.085, *p* = 0.037). The logistic regression analyses indicated that the heteroplasmic levels of m.16180_16181delAA was associated with KC (*p* < 0.005). Linear regression analyses demonstrated that the heteroplasmic levels of m.16180_16181delAA and m.302A>C were not correlated with thinnest corneal thickness (TCT), steep keratometry (Ks), and flat keratometry (Kf) (all *p* > 0.05) in KC cases and controls separately.

**Conclusion:** The current study characterized the mtDNA heteroplasmy profile in KC, and revealed that the heteroplasmic levels of m.16180_16181delAA were associated with KC.

## 1 Introduction

Keratoconus (KC) is a progressive corneal ectatic disorder characterized by corneal thinning, irregular astigmatism, and vision deterioration (Rabinowitz, 1998). The disease is typically bilateral and usually happens in adolescence (Lucas and Burdon, 2020). It is also one of the most frequent causes of corneal transplantation (Sarezky et al., 2017). Although the exact cause of KC is still unclear in most patients, the genetic, environmental, and behavioral factors were reported to be associated with its pathogenesis (Ferrari and Rama, 2020; Hao et al., 2022; Santodomingo-Rubido et al., 2022). Recently, several studies have reported that oxidative stress plays an important role in the development of KC (Navel et al., 2021; Vallabh et al., 2017), and mitochondria have been implicated in the pathogenesis of KC due to its crucial role in oxidative stress (Roy et al., 2019).

The human mitochondrial genome comprises circular double-stranded DNA that encodes 37 genes, of which thirteen genes are subunits of respiratory complexes, twenty-two genes encode mitochondrial tRNAs, and a further two encode rRNA. Generally, the mutation rate within the mitochondrial genome is much higher than the nuclear genome (Hahn and Zuryn, 2019). In addition, the mutations of mitochondrial DNA (mtDNA) could emerge as homoplasmy or heteroplasmy. The mtDNA homoplasmy represents a uniform type of mtDNA in an individual (Parakatselaki and Ladoukakis, 2021), while the mtDNA heteroplasmy represents two or more types of mtDNA simultaneously existing in the same individual (Stefano et al., 2017). A recent study reported that mitochondrial heteroplasmy is pathogenic and could lead to a greater disease burden in several diseases (Wallace and Chalkia, 2013).

Previous studies have identified multiple mtDNA variants in KC. Eighty-four mtDNA variants, including two novel frameshift mutations in the mitochondrial complex I gene, have been reported in Indian patients with KC (Pathak et al., 2011). Similarly, Abu-Amero et al. (2014b) reported ten non-synonymous mtDNA mutations in KC patients from Saudi Arabia, with one non-synonymous variant heteroplasmic. Our previous study observed an increased number of non-synonymous mtDNA variants in KC patients, though not statistically significant (Xu et al., 2021). Currently, there is a paucity of information regarding mtDNA heteroplasmy in KC from China. Therefore, we aimed to characterize the mtDNA heteroplasmy profile and explore the association of mitochondrial heteroplasmic levels with KC in the present study.

## 2 Materials and methods

### 2.1 Study population

A total of 300 KC patients (221 males and 79 females, with a mean age of 20.69 ± 4.68 years) were consecutively recruited between June 2018 and June 2021 in Henan Eye Hospital. The ratio of male to female was 2.80:1 in this study. The ratio was consistent with our previous study (Yang et al., 2021) indicating that the ratio was 2.65:1 in central China, and the study population was more representative. In addition, 300 controls without KC (224 males and 76 females, with a mean age of 20.44 ± 4.21 years) were also enrolled. KC patients were diagnosed based on the following criteria: corneal tomography revealing an asymmetric bowtie pattern with or without skewed axes or keratoconus sign detected by slit lamp examination, such as localized stromal thinning, conical protrusion, Vogt’s striae, Fleischer’s ring or anterior stromal scar (Mas Tur et al., 2017). While for the control group, the slit lamp examination showed normal cornea, and the elevation map in the tomographic map was within the normal limits. All the subjects were unrelated individuals and matched by age and sex with KC cases.

### 2.2 Clinical examination

All the subjects underwent clinical examinations. Basic characteristics were collected through medical records. The slit lamp examinations and corneal tomography measurements were performed by an experienced operator. Corneal tomography was carried out based on the Scheimplfug technique using Pentacam HR (Oculus, Wetzlar, Germany). Moreover, the thinnest corneal thickness (TCT), steep keratometry (Ks), and flat keratometry (Kf) were collected. Considering the characteristic of higher Ks in KC, the eye with a higher Ks in each subject was included in the current analysis.

### 2.3 Mitochondrial DNA sequencing and bioinformatics analysis

Total DNA was extracted from peripheral blood samples with QIAamp DNA Blood kits (Qiagen, Hilden, Germany) according to the manufacturer’s instructions. The mitochondrial genome was amplified by long-range PCR using human mitochondrial genome specific primers. The detailed procedure of mtDNA sequencing has been described in our previous article (Xu et al., 2021). After sequencing on Illumina NovaSeq System (Illumina, San Diego, CA, United States), Fastq data were obtained and aligned to the mitochondrial reference sequence (revised Cambridge Reference Sequence, rCRS) by Burroughs-Wheeler Aligner (BWA) (Li and Durbin, 2009). The quality control of the sequencing data was conducted for each sample. The mean Q20 of the samples in R1 and R2 were 0.947 ± 0.047 and 0.969 ± 0.038 separately. The mean Q30 of the samples in R1 and R2 were 0.921 ± 0.051 and 0.843 ± 0.077 separately. The Genome Analysis Toolkit (GATK) (McKenna et al., 2010) was used to identify variants. Variants with a frequency range of 0.1–0.9 were considered heteroplasmic, while variants with a frequency of ≤0.1 or a frequency of ≥0.9 were considered homoplasmic. The ratio of the mutant allele was used to represent the heteroplasmic level. As previously recommended (Cosemans et al., 2023), only heteroplasmic variants with a prevalence of at least 10% in the study population were included for further analysis.

### 2.4 Statistical analyses

Statistical analyses were performed using SPSS 21.0 (SPSS Inc., Chicago, IL, United States). Quantitative variables were expressed using mean ± standard deviation (SD) and analyzed by the Student’s t-test. The distribution of sex was analyzed by the chi-squared test. The number of heteroplasmic and homoplasmic mtDNA variants was calculated in protein-coding genes (including *ATP6*, *ATP8*, *COX1*, *COX2*, *COX3*, *CYTB*, *ND1*, *ND2*, *ND3*, *ND4*, *ND4L*, *ND5*, and *ND6*), rRNA genes, tRNA genes, and non-coding region. A comparison of heteroplasmic and homoplasmic non-synonymous variants numbers in protein-coding genes was carried out by Fisher’s exact test or chi-squared test. The Spearman’s correlation was used to demonstrate the correlation between the number of heteroplasmic variants and age. Logistic regression analysis was adopted to explore the association of mtDNA heteroplasmic level with KC, and the *β* coefficient, stand error (SE), and odds ratio (*OR*) values were recorded. An adjusted significance level of *p* = 0.05/10 = 0.005 was applied. In addition, linear regression analysis was used to explore the relationship between mtDNA heteroplasmic levels and clinical parameters, and the *β* coefficient, SE, and t values were recorded.

## 3 Results

### 3.1 Clinical characteristics of study subjects

The clinical characteristics were compared between 300 KC cases and 300 controls. As is shown in Table 1, there were no significant differences in age and sex between KC cases and controls (*p* > 0.05). The TCT in KC cases was significantly lower than that in controls (*p* < 0.05). In contrast, the Ks and Kf in KC cases were significantly higher than those in controls (*p* < 0.05).

**TABLE 1:** Clinical characteristics of the study population.

	KC (*n* = 300)	Controls (*n* = 300)	*p*
Sex (male/female)	221/79	224/76	0.780
Age (years)	20.69 ± 4.68	20.44 ± 4.21	0.486
TCT (µm)	434.16 ± 57.28	547.62 ± 28.81	<0.001*
Ks (D)	57.43 ± 11.38	43.46 ± 1.51	<0.001*
Kf (D)	52.62 ± 9.70	42.33 ± 1.40	<0.001*

TCT, thinnest corneal thickness; Ks, steep keratometry; Kf, flat keratometry.

### 3.2 Distribution of heteroplasmic and homoplasmic mtDNA variants

**FIGURE 1:**
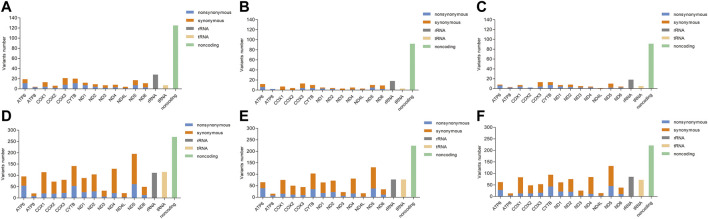
The distribution of heteroplasmic and homoplasmic mtDNA variants across the mitochondrial genome. [**(A)**: Distribution of heteroplasmic mtDNA variants in all subjects; **(B)**: Distribution of heteroplasmic mtDNA variants in KC cases; **(C)**: Distribution of heteroplasmic mtDNA variants in controls; **(D)**: Distribution of homoplasmic mtDNA variants in all subjects; **(E)**: Distribution of homoplasmic mtDNA variants in KC cases; **(F)**: Distribution of homoplasmic mtDNA variants in controls].

The distribution of heteroplasmic and homoplasmic mtDNA variants across the mitochondrial genome was shown in Figure 1. Firstly, the heteroplasmic and homoplasmic variants were calculated in all the subjects (Supplementary Table S1). A total of 311 heteroplasmic variants and 1936 homoplasmic variants were identified. The non-coding region harbored the highest (125 out of 311) heteroplasmic variants among different mitochondrial regions. While in the protein-coding genes, the *COX3* gene exhibited the highest heteroplasmic variants (Figure 1A). Then the heteroplasmic and homoplasmic mtDNA variants were calculated in KC cases and controls separately (Supplementary Tables S2, S3). In KC cases, nearly half of the heteroplasmic variants (92 out of 200) were observed in the non-coding region (Figure 1B). Similarly, 91 out of 199 variants were located in the non-coding region in the controls (Figure 1C). Among the protein-coding genes, the *COX3* gene contained 13 heteroplasmic variants in KC cases, followed by 12 heteroplasmic variants in the *ATP6* region. While in the control group, 13 heteroplasmic variants were observed in the *COX3* gene and *CYTB* gene separately, followed by 10 heteroplasmic variants in the *ND5* region.

**FIGURE 2:**
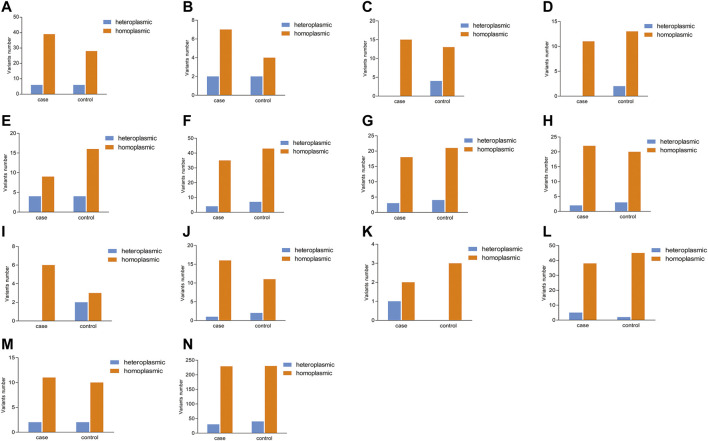
Comparison of the number of heteroplasmic and homoplasmic nonsynonymous variants in protein-coding genes between KC cases and controls. [**(A)**: *ATP6*; **(B)**: *ATP8*; **(C)**: *COX1*; **(D)**: *COX2*; **(E)**: *COX3*; **(F)**: *CYTB*; **(G)**: *ND1*; **(H)**: *ND2*; **(I)**: *ND3*; **(J)**: *ND4*; **(K)**: *ND4L*; **(L)**: *ND5*; **(M)**: *ND6*; **(N)**: Total protein-coding genes].

**FIGURE 3:**
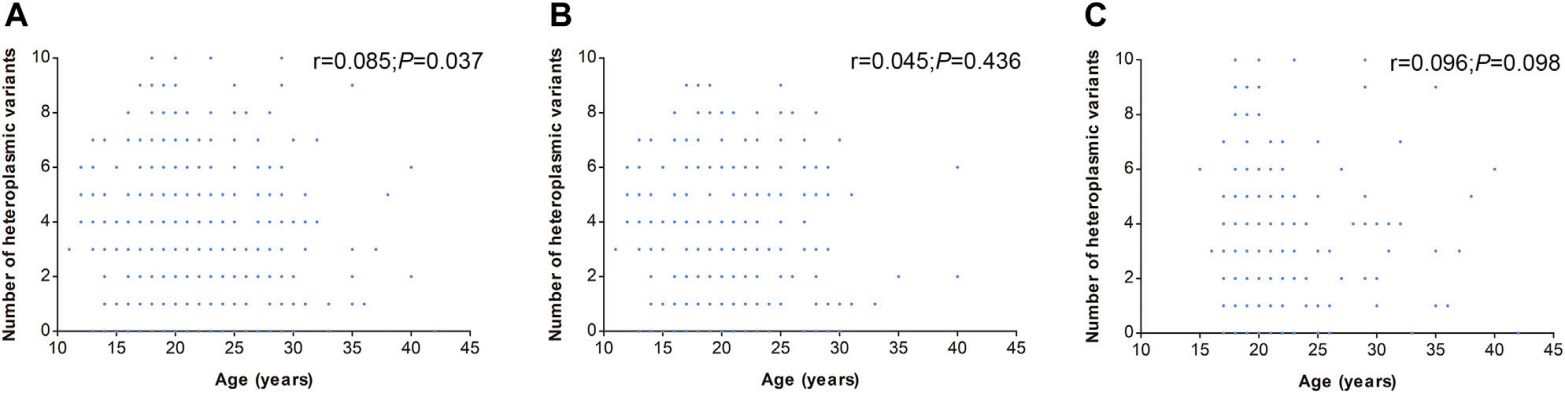
Correlation of mtDNA heteroplasmic variants number with age. [**(A)**: All subjects; **(B)**: KC cases; **(C)**: Controls].

In addition, the number of heteroplasmic and homoplasmic non-synonymous variants in protein-coding genes was compared between KC cases and controls (Figure 2; Supplementary Table S4). Finally, no statistically significant differences were found between the two groups. The correlation between the number of heteroplasmic variants and age was further analyzed in all subjects, KC cases, and controls separately (Figure 3). The number of heteroplasmic variants was significantly positively associated with age in all subjects (*r* = 0.085, *p* = 0.037), while the number of heteroplasmic variants was not associated with age in KC cases and controls separately (all *p* > 0.05).

### 3.3 Association of mtDNA heteroplasmic level with KC

The association of mtDNA heteroplasmic level with KC was explored by logistic regression with adjusting age and sex. Ten heteroplasmic variants were included in the analysis. The prevalence of the ten heteroplasmic variants in our study population was shown in Supplementary Table S5. The heteroplasmic levels of variants in KC cases and controls were shown in Supplementary Table S6, and the associations of heteroplasmic levels with KC were shown in Table 2. The results showed that the heteroplasmic level of m.16180_16181delAA were associated with KC after Bonferroni correction (*p* = 0.004, *OR* = 0.001, 95%*CI*: 0–0.097).

**TABLE 2:** Logistic regression analysis of mitochondrial heteroplasmic level and KC with adjusting age and sex.

Variant	*β*	SE	*p*	*OR* (95%*CI*)
m.302A>C	−2.546	0.981	0.009	0.078 (0.011–0.536)
m.302insCC	−0.26	1.023	0.799	0.771 (0.104–5.721)
m.302insCCC	−0.419	0.67	0.532	0.658 (0.177–2.447)
m.302insCCCC	−0.583	0.745	0.434	0.558 (0.13–2.403)
m.310T>C	0.431	1.353	0.750	1.539 (0.109–21.813)
m.16180delA	0.891	2.163	0.680	2.438 (0.035–169.000)
m.16180_16181delAA	−7.162	2.462	0.004	0.001 (0–0.097)
m.16182A>C	−0.531	1.835	0.772	0.588 (0.016–21.454)
m.16182insC	−3.995	4.242	0.346	0.018 (0–75.175)
m.16183A>C	−2.673	1.585	0.092	0.069 (0.003–1.542)

### 3.4 Correlation of mtDNA heteroplasmic level with clinical characteristics

Moreover, linear regression analyses were performed between m.302A>C,m.16180_16181delAA and clinical parameters including TCT, Ks, and Kf in KC cases and controls separately. As is shown in Table 3, the heteroplasmic levels of m.302A>C and m.16180_16181delAA were not correlated with TCT, Ks, and Kf (all *p* > 0.05).

**TABLE 3:** Liner regression analyses between heteroplasmic variants and TCT, Ks, Kf in KC cases and controls.

Parameter	KC				Control			
	*β*	SE	t	*p*	*β*	SE	t	*p*
TCT								
m.302A>C	−4.895	36.277	−0.135	0.893	6.063	16.808	0.361	0.720
m.16180_16181delAA	93.906	80.090	1.173	0.248	0.899	42.770	0.021	0.983
Ks								
m.302A>C	0.247	5.405	0.046	0.964	0.441	0.734	0.601	0.551
m.16180_16181delAA	−0.698	13.969	−0.050	0.960	−3.259	2.552	−1.277	0.209
Kf								
m.302A>C	−1.891	4.270	−0.443	0.660	0.219	0.723	0.303	0.764
m.16180_16181delAA	−7.823	11.013	−0.710	0.482	−2.877	2.300	−1.251	0.218

TCT, thinnest corneal thickness; Ks, steep keratometry; Kf, flat keratometry.

## 4 Discussion

The pathogenesis of KC is complex, and multiple studies have indicated an association of the mitochondrial genome with KC (Pathak et al., 2011; Abu-Amero et al., 2014b; Vallabh et al., 2017). In this study, we presented an extensive profile of mitochondrial heteroplasmy in KC. The results showed that the *COX3* gene harbored the highest number of heteroplasmic variants among different protein-coding genes. In addition, a positive association was found between the number of heteroplasmic variants and age in all the subjects. Logistic regression analyses indicated that the heteroplasmic level of m.16180_16181delAA was associated with KC.

KC is a corneal disorder with complex etiology. Currently, growing evidence suggests that oxidative stress may play an important role in its pathogenesis (Navel et al., 2021; Monteiro de Barros and Chakravarti, 2022). Oxidative stress refers to the imbalance of two opposite and antagonistic forces, production of reactive oxygen species (ROS) and antioxidants (Sies, 2020). Actually, mitochondria are the most important source of ROS in most mammalian cells (Hernansanz-Agustin and Enriquez, 2021). Therefore, the mitochondrial dysfunction might lead to the imbalance of oxidative stress, finally resulting in the occurrence of diseases. Recently, multiple studies indicated an association of mitochondrial genome with KC, and several mtDNA variants have been identified in KC (Atilano et al., 2005; Abu-Amero et al., 2014a). As is reported previously, most of the pathogenic mtDNA variants are heteroplasmic, and mitochondrial heteroplasmy tends to show high pathogenicity (Ye et al., 2014; Nandakumar et al., 2021). Mitochondrial heteroplasmy is the co-existence of multiple mtDNA variants in a single source, and it is now generally accepted to play important roles in many diseases (Parakatselaki and Ladoukakis, 2021). However, the profile of mitochondrial heteroplasmy in KC has not been characterized up to now. The present study elucidated the genetic profile of mitochondrial heteroplasmy in KC. A comparison of heteroplasmic variants number among different regions showed that the heteroplasmic variants were mostly located in the non-coding region, consistent with Fendt et al. (2020) who reported that most heteroplasmic variants were located in the non-coding region in oral squamous cell carcinoma. While in the protein-coding genes, the *COX3* gene exhibited the highest heteroplasmic variants. Since most mtDNA variants implicated in diseases are heteroplasmic, we inferred that the *COX3* gene might have important roles in KC. Although the heteroplasmic levels of variants in *COX3* gene exhibited no association with KC in the current study, our previous study identified an association between the *COX3* gene and KC through gene-based SKAT analysis (Xu et al., 2021). Therefore, we speculated that each variant in *COX3* gene might have a minor effect on KC, the combined effects of multiple variants resulted in the occurrence of the disease. Abu-Amero et al. (2014b) detected ten potentially pathogenic non-synonymous mtDNA mutations in KC, of which one non-synonymous variant was heteroplasmic, and the other nine were homoplasmic. Similarly, we also found a lower number of heteroplasmic non-synonymous variants in KC. However, there were no significant differences between heteroplasmic and homoplasmic non-synonymous variants. It has been reported that mtDNA heteroplasmy is associated with age (Nandakumar et al., 2021; Stewart and Chinnery, 2021). Therefore, we analyzed the correlation between the number of heteroplasmic variants and age. The results showed that the number of heteroplasmic variants was positively correlated with age in all subjects. However, there was no correlation when KC cases and controls were analyzed separately, which might be attributed to different phenotypes and sample sizes.

Different mitochondrial heteroplasmy levels have been implicated in many diseases. As is reported by Geng et al. (2019), the heteroplasmic levels in peripheral blood leukocytes were closely associated with clinical manifestations and valuable for evaluating the clinical severity of the m.3243A>G mutation. Besides, Atilano et al. (2021) found the m.13095T>C and m.13105A>G heteroplasmic levels were higher in age-related macular degeneration, with the higher heteroplasmic levels possibly representing potential biomarkers. In the present study, the association of heteroplasmic levels with KC was analyzed. The results showed that the heteroplasmic level of m.16180_16181delAA was associated with KC after adjusting the age and sex. In detail, the lower heteroplasmic level of m.16180_16181delAA owned a higher risk of KC. The m.16180_16181delAA was located in the non-coding region. Currently, multiple mtDNA variants in non-coding regions were reported to be associated with certain diseases. Deng et al. (2021) analyzed the correlation of mtDNA variants in the D-loop region with polycystic ovary syndrome, and found that the variants m.G207A, m.16036insGG, and m.16049insG were associated with decreased risk of the disease. Wu et al. (2018) identified several mtDNA variants in the D-loop region associated with Parkinson’s disease. However, the linkage of m.16180_16181delAA with other diseases hasn’t been reported. The lower TCT, higher Ks, and higher Kf were typical clinical characteristics of KC in corneal tomography (Mas Tur et al., 2017). However, the genetic correlation between those clinical parameters and the mitochondrial genome was not clear. Considering the association between the heteroplasmic levels of mtDNA variants and KC, we speculated that there might exist a correlation between the mitochondrial heteroplasmic levels and TCT, Ks, and Kf. The association analysis indicated that the heteroplasmic level of m.16180_16181delAA was associated with KC after Bonferroni correction (*p* = 0.004), and the heteroplasmic level of m.302A>C was associated with KC before correction (*p* = 0.009). Therefore, we explored the correlation between m.16180_16181delAA, m.302A>C heteroplasmic levels and TCT, Ks, Kf. Nevertheless, our findings showed that the heteroplasmic levels in m.16180_16181delAA and m.302A>C were not correlated with TCT, Ks and Kf in KC cases and controls, indicating that the differences of m.16180_16181delAA and m.302A>C heteroplasmic levels might not be attributed to TCT, Ks, and Kf. It is reported that the mitochondria dysfunction has been observed in KC. Given that the mitochondrial heteroplasmy played important roles in maintaining the mitochondrial function (Stefano et al., 2017), we inferred that the heteroplasmic levels of m.16180_16181delAA and m.302A>C might affect the risk of KC through influencing mitochondrial function. The mitochondrial dysfunction results in the change of oxidative stress, thus leading to the occurrence of KC.

Several limitations should be noted in the present study. Firstly, we only included peripheral blood samples to explore the mtDNA heteroplasmy in KC due to the difficulty of acquiring the cornea. Secondly, the mtDNA content, smoking status, and BMI were not adjusted in the analysis. Those influencing factors would be collected and analyzed in our following search. Thirdly, the mitochondrial function of heteroplasmic variants and the molecular basis of the relationship between mtDNA heteroplasmy and KC were not explored in the present study. Further studies will be conducted in the future.

In conclusion, the present study characterized the profile of mtDNA heteroplasmy in KC, and revealed that the heteroplasmic level of m.16180_16181delAA was associated with KC. The data implied that mitochondrial heteroplasmy might be involved in the pathogenesis of KC. Further research is required to better understand the complex interactions between mitochondrial heteroplasmy and KC.

## Data Availability

The datasets presented in this article are not readily available because data obtained from mitochondrial sequencing are sensitive. Requests to access the datasets can be directed to the corresponding author.
