# Supplementary material for: Mitochondrial DNA heteroplasmy analysis in keratoconus patients from China
Source: Front Genet. 2023 Sep 18;14:1251951. doi: 10.3389/fgene.2023.1251951 (PMC10544337; doi:10.3389/fgene.2023.1251951)
Supplement: Supplementary file 1 [file Table1.DOCX]

Table S1. Number of heteroplasmic and homoplasmic variants in all subjects

| Region | heteroplasmic | | |  | homoplasmic | | |
| --- | --- | --- | --- | --- | --- | --- | --- |
|  | total | nonsynonymous | synonymous |  | total | nonsynonymous | synonymous |
| *ATP6* | 19 | 11 | 8 |  | 95 | 54 | 41 |
| *ATP8* | 4 | 3 | 1 |  | 20 | 8 | 12 |
| *COX1* | 13 | 4 | 9 |  | 114 | 20 | 94 |
| *COX2* | 6 | 2 | 4 |  | 72 | 19 | 53 |
| *COX3* | 21 | 8 | 13 |  | 80 | 22 | 58 |
| *CYTB* | 20 | 11 | 9 |  | 141 | 53 | 88 |
| *ND1* | 12 | 7 | 5 |  | 88 | 25 | 63 |
| *ND2* | 9 | 4 | 5 |  | 104 | 29 | 75 |
| *ND3* | 7 | 2 | 5 |  | 32 | 6 | 26 |
| *ND4* | 8 | 3 | 5 |  | 129 | 21 | 108 |
| *ND4L* | 4 | 1 | 3 |  | 21 | 3 | 18 |
| *ND5* | 17 | 7 | 10 |  | 195 | 61 | 134 |
| *ND6* | 11 | 3 | 8 |  | 49 | 13 | 36 |
| *RNR1* | 19 | - | - |  | 43 | - | - |
| *RNR2* | 9 | - | - |  | 68 | - | - |
| tRNA | 7 | - | - |  | 115 | - | - |
| noncoding | 125 | - | - |  | 270 | - | - |

Table S2. Number of heteroplasmic and homoplasmic variants in KC cases

| Region | heteroplasmic | | |  | homoplasmic | | |
| --- | --- | --- | --- | --- | --- | --- | --- |
|  | total | nonsynonymous | synonymous |  | total | nonsynonymous | synonymous |
| *ATP6* | 12 | 6 | 6 |  | 65 | 39 | 26 |
| *ATP8* | 2 | 2 | 0 |  | 15 | 7 | 8 |
| *COX1* | 7 | 0 | 7 |  | 75 | 15 | 60 |
| *COX2* | 4 | 0 | 4 |  | 50 | 11 | 39 |
| *COX3* | 13 | 4 | 9 |  | 44 | 9 | 35 |
| *CYTB* | 10 | 4 | 6 |  | 103 | 35 | 68 |
| *ND1* | 5 | 3 | 2 |  | 64 | 18 | 46 |
| *ND2* | 4 | 2 | 2 |  | 72 | 22 | 50 |
| *ND3* | 3 | 0 | 3 |  | 22 | 6 | 16 |
| *ND4* | 5 | 1 | 4 |  | 81 | 16 | 65 |
| *ND4L* | 3 | 1 | 2 |  | 17 | 2 | 15 |
| *ND5* | 10 | 5 | 5 |  | 130 | 38 | 92 |
| *ND6* | 9 | 2 | 7 |  | 34 | 11 | 23 |
| *RNR1* | 12 | - | - |  | 34 | - | - |
| *RNR2* | 6 | - | - |  | 43 | - | - |
| tRNA | 3 | - | - |  | 77 | - | - |
| noncoding | 92 | - | - |  | 224 | - | - |

Table S3. Number of heteroplasmic and homoplasmic variants in controls

| Region | heteroplasmic | | |  | homoplasmic | | |
| --- | --- | --- | --- | --- | --- | --- | --- |
|  | total | nonsynonymous | synonymous |  | total | nonsynonymous | synonymous |
| *ATP6* | 8 | 6 | 2 |  | 62 | 28 | 34 |
| *ATP8* | 3 | 2 | 1 |  | 13 | 4 | 9 |
| *COX1* | 7 | 4 | 3 |  | 83 | 13 | 70 |
| *COX2* | 2 | 2 | 0 |  | 48 | 13 | 35 |
| *COX3* | 13 | 4 | 9 |  | 53 | 16 | 37 |
| *CYTB* | 13 | 7 | 6 |  | 94 | 43 | 51 |
| *ND1* | 7 | 4 | 3 |  | 61 | 21 | 40 |
| *ND2* | 8 | 3 | 5 |  | 75 | 20 | 55 |
| *ND3* | 5 | 2 | 3 |  | 25 | 3 | 22 |
| *ND4* | 4 | 2 | 2 |  | 84 | 11 | 73 |
| *ND4L* | 1 | 0 | 1 |  | 14 | 3 | 11 |
| *ND5* | 10 | 2 | 8 |  | 132 | 45 | 87 |
| *ND6* | 4 | 2 | 2 |  | 38 | 10 | 28 |
| *RNR1* | 12 | - | - |  | 39 | - | - |
| *RNR2* | 6 | - | - |  | 46 | - | - |
| tRNA | 5 | - | - |  | 72 | - | - |
| noncoding | 91 | - | - |  | 221 | - | - |

Table S4. Comparison of heteroplasmic and homoplasmic nonsynonymous variants in protein-coding genes between KC cases and controls

| Region | KC | |  | Control | | *P* |
| --- | --- | --- | --- | --- | --- | --- |
|  | heteroplasmic | homoplasmic |  | heteroplasmic | homoplasmic |  |
| *ATP6* | 6 | 39 |  | 6 | 28 | 0.597 |
| *ATP8* | 2 | 7 |  | 2 | 4 | 1.000 |
| *COX1* | 0 | 15 |  | 4 | 13 | 0.104 |
| *COX2* | 0 | 11 |  | 2 | 13 | 0.492 |
| *COX3* | 4 | 9 |  | 4 | 16 | 0.681 |
| *CYTB* | 4 | 35 |  | 7 | 43 | 0.772 |
| *ND1* | 3 | 18 |  | 4 | 21 | 1.000 |
| *ND2* | 2 | 22 |  | 3 | 20 | 0.96 |
| *ND3* | 0 | 6 |  | 2 | 3 | 0.182 |
| *ND4* | 1 | 16 |  | 2 | 11 | 0.565 |
| *ND4L* | 1 | 2 |  | 0 | 3 | 1.000 |
| *ND5* | 5 | 38 |  | 2 | 45 | 0.363 |
| *ND6* | 2 | 11 |  | 2 | 10 | 1.000 |
| Total | 30 | 229 |  | 40 | 230 | 0.273 |

Table S5 The prevalence of heteroplasmic variants in the study population

| Variant | prevalence in the study population (n, %) |
| --- | --- |
| m.302A>C | 93 (15.5) |
| m.302insCC | 212 (35.3) |
| m.302insCCC | 196 (32.7) |
| m.302insCCCC | 134 (22.3) |
| m.310T>C | 95 (15.8) |
| m.16180delA | 138 (23.0) |
| m.16180_16181delAA | 82 (13.7) |
| m.16182A>C | 86 (14.3) |
| m.16182insC | 61 (10.2) |
| m.16183A>C | 94 (15.7) |

Table S6. The heteroplasmic levels of variants in KC cases and controls

| Variant | KC | Control |
| --- | --- | --- |
| m.302A>C | 0.239±0.187 | 0.380±0.275 |
| m.302insCC | 0.729±0.144 | 0.731±0.127 |
| m.302insCCC | 0.369±0.205 | 0.386±0.228 |
| m.302insCCCC | 0.395±0.229 | 0.427±0.269 |
| m.310T>C | 0.698±0.179 | 0.683±0.150 |
| m.16180delA | 0.242±0.088 | 0.237±0.071 |
| m.16180_16181delAA | 0.347±0.106 | 0.423±0.105 |
| m.16182A>C | 0.444±0.141 | 0.450±0.093 |
| m.16182insC | 0.346±0.055 | 0.366±0.076 |
| m.16183A>C | 0.720±0.209 | 0.783±0.105 |
